# Ceramidas Plasmáticas na Estratificação de Risco das Doenças Cardiovasculares

**DOI:** 10.36660/abc.20201165

**Published:** 2022-04-07

**Authors:** Débora L. M. Junqueira, Alline Stach, Adriano Caixeta, Juliana Sallum, Erika Yasaki, Jeane Tsutsui, Edgar Rizatti, Carlos E. Rochitte, Ching-Jianhong, Jean-Paul Kovalik, José E. Krieger, A. Mark Richards, Mark Y. Chan, Leonardo P. de Carvalho

**Affiliations:** 1 Universidade Federal de São Paulo Escola Paulista de Medicina São Paulo SP Brasil Universidade Federal de São Paulo – Escola Paulista de Medicina, São Paulo, SP – Brasil; 2 Hospital do Coração São Paulo SP Brasil Hospital do Coração (HCor), São Paulo, SP – Brasil; 3 Grupo Fleury São Paulo SP Brasil Grupo Fleury, São Paulo, SP – Brasil; 4 Instituto Nacional do Coração Universidade de São Paulo São Paulo SP Brasil Instituto Nacional do Coração – InCor, Universidade de São Paulo, São Paulo, SP – Brasil; 5 DUKE-NUS Medical School Singapore DUKE-NUS Medical School, Singapore – Singapore; 6 Yong Loo-Lin School of Medicine National University of Singapore Singapore Yong Loo-Lin School of Medicine, National University of Singapore – Singapore

**Keywords:** Doenças Cardiovasculares, Doença da Arterial Coronariana, Ceramidas/uso terapêutico, Glicoesfingolipideos Neutros/uso terapêutico, Estratificação das Doenças, Diabetes Mellitus, Dislipidemias, Biomarcadores

## Abstract

A produção de ceramida ocorre em todo o corpo e desempenha um papel importante na manutenção da fisiologia normal. No entanto, os níveis de ceramidas são alterados em estados de doença, principalmente durante o desenvolvimento de diabetes e dislipidemia. A produção de ceramidas também está associada à instabilidade das placas ateroscleróticas. Estudos recentes revelam que pacientes com doença arterial coronariana instável apresentam níveis plasmáticos aumentados de ceramidas (principalmente C16, C18 e C24:1). Atualmente, são consideradas biomarcadores emergentes nas doenças cardiovasculares, sendo utilizadas na predição de instabilidade da placa aterosclerótica e eventos cardiovasculares adversos de forma independente aos fatores de risco tradicionais. Com o objetivo de descrever e discutir o papel das ceramidas na estratificação das doenças cardiovasculares, o desenvolvimento desta revisão narrativa contextualiza a importância desse biomarcador no cenário atual da cardiologia.

## Introdução

Dados da Organização Mundial da Saúde (OMS) demonstram que as doenças cardiovasculares (DCV) foram responsáveis por uma grande porcentagem dos 50 milhões de óbitos ocorridos na última década: cerca de 17 milhões de pessoas.^1^ Essa mortalidade é especialmente alta na fase aguda após um infarto agudo do miocárdio (IAM), com 10-15% de recorrência de eventos isquêmicos em 1 ano e taxas cumulativas de até 50% após 10 anos.^2^

Aproximadamente 50% dos pacientes submetidos a intervenção coronária percutânea primária (ICP) possuem doença multivascular que, de forma geral, apresenta-se como uma doença crônica progressiva e com altas taxas de mortalidade. Atualmente, não podemos prevenir com precisão a recorrência de eventos isquêmicos agudos, claramente demonstrando a grande necessidade de biomarcadores que possam prever a instabilidade da placa aterosclerótica coronariana.^3^

Estudos recentes têm destacado o papel fisiopatológico de outras classes lipídicas além do colesterol de lipoproteína de baixa densidade (*low density lipoprotein*, LDL) na aterosclerose e no IAM, incluindo ceramidas, esfingomielina, fosfatidilcolinas e ésteres de colesterol.^4,5^ As ceramidas participam de múltiplas vias envolvidas na sinalização de danos celulares, causando a liberação de citocinas pró-inflamatórias que modulam diretamente a apoptose por meio da expressão gênica de proteínas pró-apoptóticas.^6^

Nosso grupo tem trabalhado no desenvolvimento de biomarcadores lipídicos por meio de espectrometria de massa, entre outras técnicas de biologia molecular, para o desenvolvimento de um biomarcador plasmático capaz de diagnosticar a instabilidade da placa aterosclerótica e prever reinfartos cardíacos e a progressão para insuficiência cardíaca (IC) em pacientes com síndrome coronariana aguda (SCA).

A ceramida é um biomarcador lipídico com papel emergente no diagnóstico precoce e na estratificação de risco, atuando como biomarcador de eventos cardiovasculares (CVs) primários e secundários em pacientes com aterosclerose clínica e subclínica suscetíveis ao desenvolvimento de eventos isquêmicos agudos.^5,7^

### Ceramida: uma breve revisão da fisiologia desse novo biomarcador lipídico.

As ceramidas e o colesterol LDL são lipídios estruturais da membrana que mantêm sua fluidez e integridade por meio da formação de poros seletivos, modulando a movimentação de compostos entre os espaços intra e extracelulares. A ceramida é um esfingolipídio formado pela associação de uma esfingosina com um ácido graxo, sendo um componente-chave na formação das membranas celulares8 e atuando como um importante intermediário na sinalização de processos que regulam a homeostase celular, tais como inflamação, apoptose e a resposta celular ao estresse.^9^

**Figura 1:**
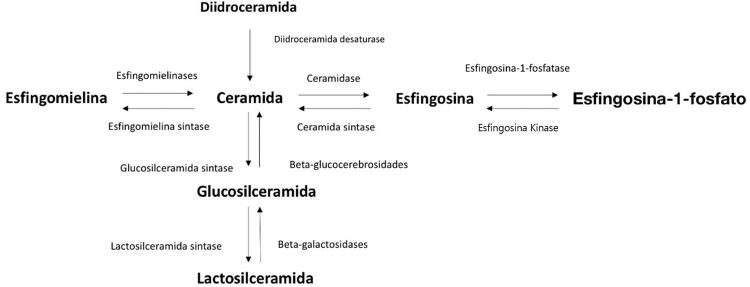
– Vias do metabolismo de esfingolipídios.

As ceramidas se acumulam na placa de ateroma coronariana^10^ e em suas formas glicosiladas: as glicosilceramidas e as lactosilceramidas (Figura 1), que são abundantes na placa em desenvolvimento.^11,12^ Além disso, dados do nosso grupo mostraram que o próprio tecido miocárdico pode produzir ceramidas diretamente em resposta à isquemia e reperfusão.^13^

A ceramidase é uma enzima que quebra os ácidos graxos da ceramida para produzir esfingosina, a qual, por sua vez, é fosforilada pela esfingosina-1-fosfatase para formar esfingosina-1-fosfato. A síntese das ceramidas pode ocorrer através da hidrólise da esfingomielina (degradação), através da via de recuperação (reciclagem) na qual a esfingosina é fosforilada ou através de uma via de novo, na qual as diidroceramidas são dessaturadas.^9^

### Ceramidas: uma ligação entre aterosclerose, diabetes e dislipidemia

**Figura 2:**
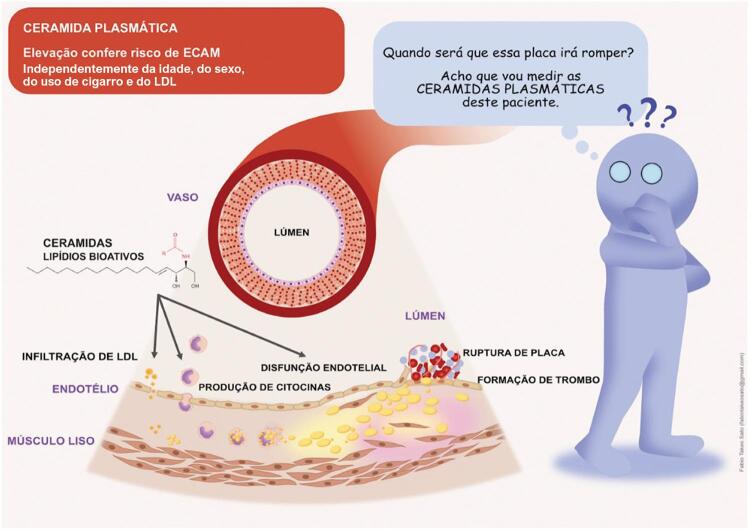
– Ceramidas plasmáticas e ruptura de placa. ECAM = eventos cardiovasculares adversos maiores. Fonte: coleção do autor.

As ceramidas constituem aproximadamente 30% do colesterol LDL circulante. O aumento da concentração de ceramida altera a permeabilidade da membrana celular, facilitando o acúmulo de colesterol LDL na parede do vaso. Esse acúmulo amplifica o processo inflamatório da parede do vaso e promove a apoptose das células musculares lisas vasculares e a disfunção endotelial, ocasionando a instabilidade da placa aterosclerótica e sua ruptura^14^ (Figura 2).

**Figura 3:**
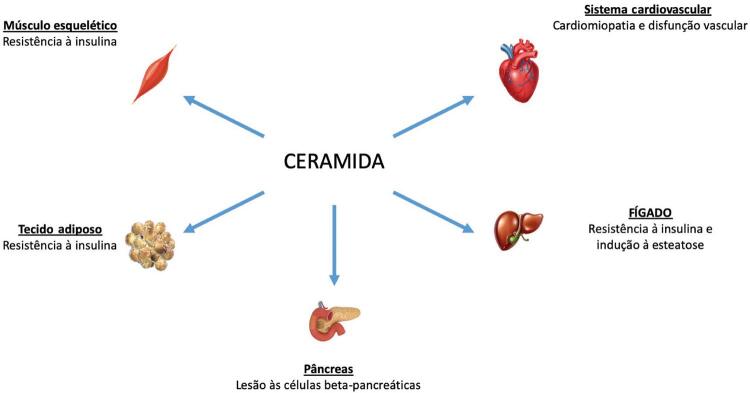
– Ação das ceramidas em diferentes órgãos.

Além da placa aterosclerótica, o acúmulo de colesterol também ocorre na musculatura lisa esquelética, interferindo na expressão do transportador de glicose tipo 4, o que ocasiona defeito no transporte muscular de glicose e prejudica sua capacidade de síntese de glicogênio.^15^ As ceramidas também estimulam a apoptose das células β pancreáticas, reduzindo diretamente a produção de insulina.^16^ Major et al.,^17^ demonstraram que as ceramidas podem mimetizar os efeitos citotóxicos do fator de necrose tumoral, da interleucina 1 beta e do interferon-gama nas células β pancreáticas, desencadeando inflamação e apoptose.^17^ O acúmulo de ceramida nos tecidos resulta na disfunção metabólica de múltiplos órgãos e no desenvolvimento de complicações do diabetes. A Figura 3 demonstra os principais tecidos afetados pelas ceramidas.^18,19^

Portanto, além de determinar o grau de dislipidemia e o risco de aterosclerose através do valor do colesterol LDL, a dosagem da ceramida também auxilia o médico a determinar a resistência insulínica e a apoptose de células beta pancreáticas.

A implicação clínica da ceramida levou à patente desse biomarcador nos EUA e na Europa, estando atualmente disponível para uso clínico em hospitais de referência, como a Mayo Clinic.^20^

### Os exames de rastreamento são necessários para o acompanhamento da aterosclerose subclínica?

Há uma lacuna significativa na detecção da doença aterosclerótica subclínica e na triagem e no acompanhamento custo-efetivos dessa condição, com muitos testes não invasivos sendo solicitados rotineiramente em populações com diferentes níveis de risco CV. Isso ocorre em todo o mundo e, apesar do avanço crescente dos testes não invasivos para a detecção da doença aterosclerótica, a estratificação de risco permanece imperfeita.^21^ Considerando os custos crescentes com os cuidados de saúde, essa prática médica estereotipada deve, portanto, ser reavaliada.

A associação de dados clínicos e de imagem no apoio ao desenvolvimento de protocolos que incorporem escores de risco clássicos, como os escores de Framingham e INTERHEART, tem sido testada com o intuito de aprimorar a estratificação de risco CV. No entanto, esses métodos, quando aplicados à população em geral sem uma etapa de triagem, têm capacidade limitada na avaliação do risco CV por razões logísticas e de custo.^22^

O *Multi-Ethnic Study of Atherosclerosis* (MESA), um dos estudos mais relevantes na área, trouxe contribuições importantes para a compreensão do desenvolvimento e da progressão da DCV do estágio subclínico ao clínico. Em uma subanálise do MESA, um grande painel de biomarcadores de proteínas foi avaliado para encontrar marcadores preditivos de progressão de DCV. Em comparação aos fatores de risco CV clássicos, biomarcadores de proteína com vários painéis específicos de inflamação, resistência à insulina, lipídios, hemostasia, fibrinólise, dano oxidativo, estresse endotelial, entre outros, foram testados e apresentaram apenas um valor preditivo incremental limítrofe para eventos adversos CVs em longo prazo (AUC: 0,768 vs. 0,776, p = 0,003) e não apresentaram valor incremental na predição de eventos CVs adversos em médio prazo (AUC: 0,795 vs. 0,796, p = 0,627). Portanto, devido aos valores semelhantes da curva característica de operação do receptor (*receiver operating characteristic*, ROC), novos métodos de rastreamento e estratificação de risco são necessários para melhorar a detecção precoce da instabilidade da placa.^23^

O desenvolvimento desse biomarcador possibilitaria a intervenção na progressão inicial da placa aterosclerótica, sendo, portanto, essencial para evitar o enorme custo adicional imposto pela fase sintomática da doença aterosclerótica. Essas considerações são importantes e devem ser avaliadas no desenvolvimento de plataformas de rastreamento de saúde populacional, objetivando uma boa relação de custo-efetividade.

### Devemos dosar as ceramidas e o colesterol LDL?

A aterosclerose subclínica antecede a maioria dos eventos CVs e sua detecção pode melhorar a estratificação de risco CV.^11,12^ No entanto, uma incompatibilidade tem sido relatada entre perfis de fatores de risco convencionais aparentemente benignos e a presença de aterosclerose subclínica detectada por calcificação da artéria coronária ou medição da espessura da íntima no ultrassom de carótidas.^13,14^

Estudos identificaram a presença de aterosclerose subclínica em quase 60% dos indivíduos de meia-idade classificados como de baixo risco CV de acordo com os escores de risco tradicionais; em 41% desses indivíduos, observaram-se múltiplos locais vasculares afetados.^15^ Esses achados sugerem que outras variáveis além dos fatores de risco CV convencionais podem desempenhar um papel relevante na aterogênese.

Os pacientes com doença aterosclerótica são uma população muito heterogênea, com estratificação de risco complexa, sendo errôneo considerar todos esses pacientes com risco semelhante de eventos agudos. Atualmente, o uso de escores de risco CV é indicado pelas diretrizes como uma ferramenta de estratificação; no entanto, devido às limitações na precisão preditiva, principalmente em pacientes com alto risco CV, a otimização das ferramentas de risco por meio da recalibração de escores ou da associação com biomarcadores é frequentemente necessária para uma maior acurácia preditiva em diferentes populações.

O colesterol LDL é um fator de risco diretamente envolvido no desenvolvimento da placa aterosclerótica e, portanto, um importante alvo terapêutico na prática clínica. Dieta, mudanças no estilo de vida e medicamentos podem resultar em reduções significativas e sustentadas do colesterol LDL plasmático. No entanto, apesar disso, as DCVs continuam sendo uma das principais causas de morte em todo o mundo,^24^ sugerindo que o controle convencional do colesterol LDL não é suficiente. A detecção e a prevenção precoces da instabilidade da placa aterosclerótica podem abrir caminhos para reduzir significativamente a progressão da doença.

As diretrizes atuais endossam o controle do colesterol LDL e a medição de marcadores inflamatórios inespecíficos, como a proteína C reativa, para a estratificação de risco CV. No entanto, a fisiopatologia da aterosclerose envolve a interseção complexa de dislipidemia, inflamação, disfunção endotelial e ativação plaquetária, entre outros fatores.^25^ Dados recentes demonstram possíveis associações entre cada uma dessas vias com os níveis plasmáticos das ceramidas, indicando associação e causalidade plausíveis desse biomarcador em DCV aguda e instabilidade da placa aterosclerótica.

Além disso, o colesterol LDL plasmático não prediz com precisão eventos cardíacos adversos maiores e eventos cerebrovasculares (*major adverse cardiac and cerebrovascular events*, MACCE), exigindo, na prática clínica, avaliação médica criteriosa e uma série de testes não invasivos para acompanhar a progressão da placa aterosclerótica.26 Por outro lado, as concentrações plasmáticas de ceramidas são elevadas em várias condições relacionadas às doenças cardíacas, além do papel bioquímico na progressão da aterosclerose, também estudado por nosso grupo anteriormente. Observamos em estudos preliminares a liberação incremental de ceramidas na fase aguda do IAM e em placas humanas instáveis, correlacionando esses achados com dados pré-clínicos que mostraram a suprarregulação de enzimas produtoras de ceramidas no miocárdio, com aumento dos níveis plasmáticos nas primeiras 24 horas após um evento isquêmico agudo.^5^

As ceramidas também estão associadas a um maior risco de evolução em pacientes com IC. Em 423 pacientes com IC aguda, as concentrações plasmáticas de ceramidas foram independentemente associadas à morte e à piora da função ventricular esquerda durante a hospitalização.^27^

### A busca por evidências: ceramidas e predição de risco CV

#### Prevenção primária:

O estudo FINRISK incluiu pacientes sem eventos cardiovasculares prévios e mostrou que os níveis circulantes de ceramidas específicas (16:0, 18:0 e 24:1) estão significativamente associados a eventos cardiovasculares maiores subsequentes quando comparados a indivíduos que permaneceram assintomáticos. As associações univariadas significativas entre ceramidas e eventos fatais sugerem que elas desempenham um papel fundamental na ruptura das placas ateroscleróticas nessa população.^7^

Além disso, Petterson et al.,^28^ demonstraram, ainda no contexto da prevenção primária, que a proporção de ceramidas C24:0/C16:0 e a concentração plasmática de ceramida C24:0 estão inversamente associadas a fatores de risco coronariano, como idade e tabagismo, e inversamente associadas ao desenvolvimento de DAC e IC.^28^

#### Doença arterial coronária:

Em uma análise post-hoc do *Systolic Blood Pressure Intervention Trial* (N = 9.631), Nguyen et al.,^31^ demonstraram que os níveis plasmáticos de colesterol LDL não estavam associados ao desfecho composto primário (infarto do miocárdio, acidente vascular cerebral, insuficiência cardíaca descompensada aguda e morte por causas CVs).^29^ Além disso, avaliando os pacientes em prevenção secundária de eventos CVs (N = 1.562), observou-se que o colesterol LDL foi apenas marginalmente associado à incidência de eventos CVs: taxa de risco ajustada: 1,005 (intervalo de confiança de 95% [IC95%] = 1,002-1,009; p = 0,005 [aumento de 1 mg/dL]), com baixa capacidade de discriminação de eventos cardiovasculares adversos maiores (MACE) (área ROC de 0,54; p = 0,087).^29^

Embora estudos com inibidores de PCSK9, como o *Odyssey Outcomes e Fourier*,^30^ reforcem o princípio “quanto menor, melhor”, significando que há uma associação entre níveis baixos de colesterol LDL e baixo risco de aterosclerose clínica, não há uma correlação de risco perfeita. Para preencher essa lacuna, o uso de métodos de medição de metabólitos por técnicas metabolômicas tem sido cada vez mais utilizado, pois esses métodos apresentam vantagens sobre os métodos clássicos devido à análise mais abrangente dos metabólitos e à capacidade de obtenção do perfil metabólico do tecido-alvo da doença de interesse.

Uma análise metabolômica não direcionada identificou três ceramidas plasmáticas significativamente associadas à mortalidade cardiovascular em uma coorte com DAC confirmada por angiografia. As ceramidas associadas à alta mortalidade cardiovascular nesse estudo foram: C16:0, C18:0 e C24:1. A associação ocorreu independentemente de idade, índice de massa corporal, tabagismo, uso de estatinas, triglicerídeos, LDL e colesterol total.^14^

Kaasenbrood et al.,^31^ tentaram melhorar a predição de eventos agudos nesse grupo de pacientes através do escore de risco SMART (manifestações secundárias de doença arterial).^31^ Esse escore de risco compreende variáveis clínicas e laboratoriais (colesterol total, colesterol HDL, taxa de filtração glomerular estimada, proteína C reativa) e foi testado em várias coortes. Com base nos resultados obtidos, os autores sugeriram novos algoritmos de estimativa de risco CV para estratificar essa população individualmente e com maior precisão, demonstrando a capacidade de aprimoramento dos escores de risco pela incorporação de biomarcadores.^31^

No *Mediterranean Diet Prevention* (PREDIMED), um estudo de coorte prospectivo em pacientes com alto risco CV, ceramidas C24:0, C22:0 e C16:0 foram associadas a DCV.4 A razão de chance (odds ratio, OR) comparando os quartis extremos das concentrações plasmáticas de ceramidas C16: 0, C22: 0, C24: 0 e C24: 1 foram de 2,39 (1,49-3,83; p < 0,001), 1,91 (1,21-3,01; p = 0,003), 1,97 (1,21-3,01; p = 0,004) e 1,73 (1,09-2,74; p = 0,011), respectivamente. Em um estudo prospectivo de aproximadamente 500 pacientes submetidos a angiografia coronária eletiva, Meeusen et al. relataram que os níveis plasmáticos de C16:0, C18:0 e C24:1 foram independentemente associados a um risco aumentado de MACCE em um acompanhamento médio de 4 anos.^32^ O risco associado às ceramidas foi novamente independente dos fatores de risco tradicionais, incluindo idade, sexo, índice de massa corporal, tabagismo e colesterol. Além disso, o valor preditivo permaneceu significativo após ajustes adicionais para glicose sérica e história familiar de DAC. Esses resultados sugerem que, quando as ceramidas plasmáticas estão altas em pacientes com ou sem estenose significativa da artéria coronária, o risco de morte é alto em ambos os grupos.^32^

Outro escore de risco envolvendo ceramidas é o CERT2, que foi desenvolvido no estudo *The Western Norway Coronary Angiography Cohort* (WECAC) e validado nos estudos Intervenção a Longo Prazo com Pravastatina em Doença Isquêmica (LIPID) e Langzeiterfolge der KARdiOLogischen Anschlussheilbehandlung (KAROLA).^33^ Os resultados mostraram que a ferramenta de estimativa de risco CV desenvolvida com a incorporação da medição de ceramidas pode estratificar os MACE de forma confiável em pacientes com DAC estável. Evidências adicionais obtidas nos estudos WECAC e LIPID demonstraram que esses biomarcadores isoladamente estratificam de forma acurada o risco CV primário em pacientes com e sem diabetes; em indivíduos com diabetes, os únicos preditores com valor significativo no estudo WECAC foram o escore CERT2 e a troponina altamente sensível.

#### SCA

No estudo *European Collaborative Project on Inflammation and Vascular Wall Remodeling in Atherosclerosis – Intravascular Ultrasound* (N = 600 pacientes), Cheng et al. demonstraram que os níveis plasmáticos de C16:0, C18:0 e C24:1 foram significativamente associados à morfologia vulnerável da placa coronária em indivíduos com SCA. Além disso, os níveis plasmáticos mais elevados dessas ceramidas também foram significativamente associados a uma maior gravidade angiográfica de estenose coronariana,^14,34^ bem como a uma menor perfusão da parede miocárdica pós-estresse em pacientes com DAC estabelecida ou suspeita que realizaram cintilografia de perfusão miocárdica.^35,36^ Esses achados sugerem causalidade entre a elevação das ceramidas na placa aterosclerótica e sua instabilidade ou gravidade.

Recentemente, Pan, Dong, Sun et al. utilizando tomografia de coerência óptica (optical coherence tomography, OCT) em pacientes com STEMI e ruptura de placa, observaram níveis plasmáticos elevados de C16:0, C18:0 e C24:0 em comparação a indivíduos sem doença coronariana e pacientes com DAC estável (p < 0,001, p < 0,001, p < 0,001, p < 0,001, respectivamente). Esse foi o primeiro estudo utilizando OCT que provou uma associação positiva e independente entre as concentrações plasmáticas de ceramidas e a presença de ruptura de placa, sugerindo que as concentrações plasmáticas de ceramidas podem atuar como potenciais biomarcadores para a ruptura de placa.^37^

Mais evidências dessa associação foram obtidas no estudo de Laaksonen et al.,^5^ Em uma coorte prospectiva de pacientes com DAC estável, 81 dos 1.580 pacientes (Tabela 1) apresentaram elevação de ceramida sérica e, posteriormente, apresentaram MACE ao longo de 4,6 anos de acompanhamento. Essa proporção foi mantida mesmo após o ajuste para o tratamento com estatinas. As ceramidas foram preditivas em ambos os casos, com OR praticamente igual em pacientes com ou sem estatinas: 1,68 (1,31-2,15) vs. 1,7 (1,33-2,17), respectivamente. Nesse estudo, o nível plasmático de colesterol LDL não foi significativamente preditivo de MACE.^5^

**Tabela 1:** – Escore de risco relativo envolvendo ceramidas em diferentes coortes

Escore	Categoria	BECAC (Risco 5 anos)^5^			SPUM-ACS (Risco 1 ano)^5^		

		Morte (n)	%	Risco Relativo	Morte (n)	%	Risco Relativo
0-2	Baixo	15/549	2,7%	1,0	9/575	1,6%	1,0
3-6	Moderado	29/601	4,8%	1,8	16/611	2,6%	1,7
7-9	Alto	20/288	6,9%	2,5	9/270	3,3%	2,1
10-12	Muito alto	17/149	11,4%	4,2	17/181	9,4%	6,0

*Fonte: adaptado da Mayo Clinic. https://www.mayoclinic.org/*

Laaksonen et al.,^5^ também analisaram o estudo SPUM-SCA (N = 1.637), realizado em pacientes com SCA, em que as ceramidas foram novamente preditoras de MACE independentemente do risco CV. Em 51 pacientes que morreram em até um ano após um evento cardíaco, as ceramidas plasmáticas mensuradas apresentaram níveis significativamente maiores em comparação aos pacientes que sobreviveram durante o acompanhamento.^5^

Finalmente, De Carvalho et al.,^13^ avaliaram pacientes com IAM em duas coortes de pacientes submetidos a estratificação invasiva, comparando as taxas de sobrevida livre de MACCE em pacientes de alto risco definidas pelo escore *Global Registry of Acute Coronary Events *(GRACE) ajustado para a população local. Nesse estudo, evidenciou-se que o escore GRACE apresentou menor capacidade de predição de sobrevida livre de eventos quando comparado ao desempenho preditivo de uma associação de 12 ceramidas plasmáticas medidas na fase aguda do IAM.^13^ Esse estudo incluiu chineses, malaios e indianos, etnias que representam uma grande proporção da população mundial. Ainda, foi realizada revalidação externa do valor preditivo desses biomarcadores em uma população de caucasianos da Nova Zelândia, demonstrando o desenvolvimento de um biomarcador com potencial uso universal. Esses dados foram ainda corroborados com análise de biologia molecular de biópsias de placas ateroscleróticas de pacientes submetidos a cirurgia cardíaca com e sem infarto recentes, confirmando o aumento da produção de ceramidas em pacientes com placa aterosclerótica instável.

Os principais estudos que avaliaram a associação entre as ceramidas e o risco de eventos CV agudos estão demonstrados na Tabela 2.

**Tabela 2:** – Principais estudos que avaliaram a associação entre as ceramidas e o risco de eventos cardiovasculares agudos (publicação ordenada por ano)

Autor/referência	Característica do estudo	Desfecho primário	Ajuste	Resultado principal
Laaksonen et al.^5^ European Heart Journal 2016;37, 1967-1976	Estudo de coorte prospectivo com N = 1580 adultos (62 anos; 59% homem; IMC 25 kg/m2; LDL 2,8 mmol/L, triglicerídeos 1,4 mmol/L; 62,6% utilizando estatina) submetidos a angiografia coronária eletiva devido a DAC estável e recrutados no Hospital da Universidade de Haukeland, em Bergen (estudo BECAC). Seguimento de 4,6 anos em associação a 1.637 pacientes (63 anos; 78% sexo masculino, IMC 26 kg/m2, LDL 2,6 mmol/L, triglicerídeos 1 mmol/L, 27,2% em uso de estatina) com diagnóstico de SCA submetidos a tratamento invasivo em quatro hospitais universitários suíços (estudo SPUM-ACS), com seguimento de 1 ano.	Morte cardiovascular	Colesterol total, triglicerídeos, colesterol HDL, colesterol LDL, idade, sexo, tabagismo, infarto agudo do miocárdio prévio, diabetes, hipertensão e acidente vascular cerebral prévio.	Cer (d18: 1/16: 0) e Cer (d18: 1/24: 1) foram associadas a um risco aumentado de morte cardiovascular em todas as coortes. OR Cer (d18:1/16:0)/Cer(d18:1/24:0) foi de 4,49 (IC95%, 2,24-8,98), 1,64 (1,29-2,08) e 1,77 (1,41-2,23) para Corogene, SPUM-ACS e estudo BECAC, respectivamente.
Havulinna et al.^7^ Arteriosclerer Thromb Vasc Biol 2016;36: 2424-2430	Estudo de coorte populacional com N = 8.101 pacientes saudáveis (idade 48 anos; 47% homens, IMC 26 kg/m2, colesterol LDL 3,3 mmol/L, triglicerídeos 1,3 mmol/L) do FINRISK 2002.	MACCE	Colesterol total, colesterol HDL, pressão arterial, diabetes melito e tabagismo.	Cer (d18: 1/16: 0), Cer (d18: 1/18: 0) e Cer (d18: 1/24: 1) foram significativamente maiores em pacientes com evolução cardiovascular adversa quando comparados a indivíduos assintomáticos. As concentrações séricas das ceramidas de alto risco que previam morte cardiovascular em pacientes com DAC também foram maiores nos casos do FINRISK MACE em comparação a indivíduos assintomáticos da seguinte forma: Cer (d18: 1/16: 0), Cer (d18: 1/18: 0) e Cer (d18: 1/24: 1) 11,4%, 21,3% e 17,0% (p < 0,001 para todos).
Wang et al.^4^ Circulation 2017; 135: 2028-2040	Estudo de coorte aninhado no estudo randomizado Mediterranean Diet Prevention, com N = 980 participantes (68 anos; 45% homens, IMC 30 kg/m2, colesterol LDL 3,4 mmol/L, triglicerídeos 1,6 mmol/L), incluindo 230 casos de DCV e 787 participantes selecionados aleatoriamente. A subcoorte incluiu 37 casos sobrepostos de DCV. Foram excluídos dois participantes com concentrações indetectáveis de ceramida no plasma. Acompanhamento: 4,5 anos.	MACE	Idade, sexo, IMC, histórico familiar de doença arterial coronariana precoce, tabagismo, história de hipertensão, dislipidemia e diabetes tipo II.	Entre as ceramidas de alto risco identificadas, os últimos quartis dos níveis plasmáticos de Cer (d18: 1/16: 0), Cer (d18: 1/22: 0), Cer (d18: 1/24: 0) e Cer (d18: 1/24: 1) foram associados a um desfecho cardiovascular adverso. A RR multivariável comparando os quartis extremos das concentrações plasmáticas de C:16, C22:0, C24:0 e C24:1 foi de 2,39 (1,49-3,83, p < 0,001), 1,91 (1,21-3,01, p = 0,003), 1,97 (1,21-3,01, p = 0,004), e 1,73 (1,09-2,74, p = 0,011), respectivamente.
De Carvalho et al.^13^ JACC Basic Transl Sci 2018;3:163-175	Estudo prospectivo e longitudinal com N = 327 pacientes de coorte primária (57 anos; 90% sexo masculino; IMC 26 kg/m2; colesterol LDL 3,1 mmol/L; triglicerídeos 1,2 mmol/L) e 119 pacientes na coorte de validação (66 anos; 72% sexo masculino; IMC 29 kg/m2; colesterol LDL 3,2 mmol/L) com SCA submetidos a estratificação invasiva com mensurações plasmáticas realizadas em dois momentos (antes e após a estratificação invasiva) com seguimento de 1 ano.	MACCE	GRACE	Entre as ceramidas de alto risco previamente identificadas, os níveis plasmáticos de Cer (d18: 1/16: 0), Cer (d18: 1/18: 0) e Cer (d18: 1/24: 1) foram associados a eventos cardiovasculares adversos.
Meeusen et al.^32^ Arterioscler Thromb Vasc Biol. 2018; 38: 1933-1939	Estudo transversal: 495 participantes (60 anos; 62% sexo masculino; IMC 28 kg/m2; colesterol LDL 3,1 mmol/L; triglicerídeos 1,7 mmol/L; uso de estatina 28,5%) antes da angiografia coronária não urgente. Acompanhamento: 4 anos.	MACE (infarto do miocárdio, intervenção percutânea, cirurgia de revascularização miocárdica, acidente vascular cerebral ou morte)	Idade, sexo, IMC, hipertensão, tabagismo, colesterol LDL, colesterol HDL, triglicerídeos, glicemia, histórico familiar de doença arterial coronariana	Entre as ceramidas de alto risco identificadas anteriormente, os níveis plasmáticos de Cer (d18: 1/16: 0), Cer (d18: 1/18: 0) e Cer (d18: 1/24: 1) foram associados a eventos cardiovasculares adversos. As RRs ajustadas para o desvio padrão (IC95%) foram 1,50 (1,16-1,93) para Cer (16: 0), 1,42 (1,11-1,83) para Cer (18: 0) e 1,43 (1,08-1,89) para Cer (24: 1)
Peterson et al.^28^ J Am Heart Assoc. 2018;7: e007931	Estudo baseado na comunidade: 2.642 participantes do Framingham Heart Study (66 anos; 46% homens; IMC 28 kg/m2, colesterol LDL 2,7 mmol/L, triglicerídeos 1,3 mmol/L, 42,7% estatina) e 3.134 participantes do Estudo de Saúde na Pomerânia (idade 54 anos; 48% homens; IMC 28 kg/m2; colesterol LDL 5,5 mmol/L; triglicerídeos 1,8 mmol/L; 14,5% em uso de estatina) foram seguidos por 6 e 8 anos, respectivamente.	MACE (i.e., eventos cardiovasculares fatais e não fatais).	Idade, sexo, índice de massa corporal, hipertensão, diabetes melito, tabagismo, anti-hipertensivos, proporção de colesterol total/HDL, triglicerídeos e medicamentos hipolipemiantes.	Entre as ceramidas de alto risco identificadas previamente, apenas as Cer (d18: 1/24: 0) foram associadas a desfechos cardiovasculares adversos. Na metanálise das duas coortes e após o ajuste dos fatores de risco para DAC, as proporções de ceramida C24: 0/C16: 0 foram inversamente associadas a DAC (RR por incremento médio do DP: 0,79; IC95%, 0,71-0,89; P < 0,0001) e inversamente associado a IC (RR: 0,78; IC95%, 0,61-1,00; P = 0,046).
Hilvo et al.^33^ European Heart Journal 2019, in press	Estudo longitudinal: três grandes estudos de coorte com 3.789 pacientes (62 anos; 72% sexo masculino; colesterol LDL 2,9 mmol/L; triglicerídeos 1,5 mmol/L; 72,6% uso de estatina) da WECAC; 5.991 pacientes (65 anos; 83% homens; colesterol LDL 3,9 mmol/L; triglicerídeos 1,6 mmol/L; 49,9% em uso de estatina) do estudo LIPID; e 1.023 pacientes (idade 62 anos; homens 84%; colesterol LDL 3 mmol/L; triglicerídeos 1,6 mmol/L; uso de estatina 75,6%) do KAROLA. Acompanhamento: 6 anos.	MACE (por exemplo, desfecho composto de morte por CV, IM e acidente vascular cerebral)	Idade, sexo, tratamento com estatinas (WECAC, KAROLA), diabetes melito, hipertensão, tabagismo atual, IM prévio, acidente vascular cerebral prévio, estratificado por intervenção com vitamina B (WECAC) e grupo de tratamento (LIPID).	Um escore de risco simples, com base nas ceramidas e fosfatidilcolinas que apresentam as melhores características prognósticas, foi desenvolvido no estudo WECAC e validado nas outras duas coortes. Essa pontuação foi altamente significativa na previsão da mortalidade por DCV [As RRs multiajustadas (IC95%) pelo DP foram 1,44 (1,28-1,63) no WECAC, 1,47 (1,34-1,61) no estudo LIPID e 1,69 (1,31- 2,17) no KAROLA. Além disso, uma combinação do escore de risco com a troponina T de alta sensibilidade aumentou as RRs para 1,63 (1,44-1,85) e 2,04 (1,57-2,64) nas coortes WECAC e KAROLA, respectivamente.

*BECAC: Bergen Coronary Angiography Cohort; DAC: doença arterial coronariana; FINRISK: population-based risk factor survey ; IC95%: intervalo de confiança de 95%; KAROLA: Langzeiterfolge der KARdiOLogischen Anschlussheilbehandlung; LIPID: Intervenção a Longo Prazo com Pravastatina em Doença Isquêmica; MACCE: eventos adversos cardíacos e cerebrovasculares maiores; MACE: eventos adversos cardíacos maiores; RR: razão de risco; SPUM-ACS: Special Program University Medicine-Inflammation in Acute Coronary Syndromes ; WECAC: The Western Norway Coronary Angiography Cohort.*

## Conclusão

As ceramidas plasmáticas são elevadas em pacientes com MACCE, e estudos pré-clínicos e clínicos demonstram uma associação desses lipídios com o processo de instabilidade da placa aterosclerótica.

A aferição das ceramidas possui valor incremental para a estratificação de risco, além dos fatores de risco clássicos, tanto na prevenção CV primária quanto na secundária. Medições consecutivas podem apresentar um valor preditivo incremental maior do que outros biomarcadores para a previsão de eventos adversos futuros. No entanto, precisamos de mais evidências obtidas através de estudos randomizados para avaliar o impacto prognóstico e do escalonamento terapêutico guiado pelos níveis plasmáticos de ceramidas.
