# Supplementary material for: Ceramidas Plasmáticas na Estratificação de Risco das Doenças Cardiovasculares
Source: Arq Bras Cardiol. 2022 Apr 7;118(4):768–77. [Article in Portuguese] doi: 10.36660/abc.20201165 (PMC9007014; doi:10.36660/abc.20201165)
Supplement: Translation

# Translation: Plasma Ceramides in Cardiovascular Disease Risk Stratification

**Authors:** Débora L. M. Junqueira, Alline Stach, Adriano Caixeta, Juliana Sallum, Erika Yasaki, Jeane Tsutsui, Edgar Rizatti, Carlos E. Rochitte, Ching-Jianhong, Jean-Paul Kovalik, José E. Krieger, A. Mark Richards, Mark Y. Chan, Leonardo P. de Carvalho

**Affiliations:** 1 Brazil Universidade Federal de São Paulo – Escola Paulista de Medicina, São Paulo, SP – Brazil; 2 Brazil Hospital do Coração (HCor), São Paulo, SP – Brazil; 3 Brazil Grupo Fleury, São Paulo, SP – Brazil; 4 Brazil Instituto Nacional do Coração – InCor, Universidade de São Paulo, São Paulo, SP – Brazil; 5 Singapore DUKE-NUS Medical School, Singapore - Singapore; 6 Singapore Yong Loo-Lin School of Medicine, National University of Singapore – Singapore

**Keywords:** Cardiovascular Diseases, Coronary Artery Disease, Ceramides/therapeutic use, Diseases Stratification, Diabetes Mellitus Dyslipidemias, Biomarkers

## Abstract

Ceramide production takes place throughout the body and plays a key role in the maintenance of normal physiology. However, ceramide levels are altered during disease states, particularly considering the development of diabetes and dyslipidemia. Ceramide production is also associated with atherosclerotic plaque instability. Recent studies revealed that patients with unstable coronary artery disease (CAD) presented increased plasma ceramide levels (especially C16, C18, and C24:1). These molecules are currently considered emerging biomarkers of cardiovascular diseases (CVD), being used for predicting atherosclerotic plaque instability and adverse cardiovascular events independently from traditional risk factors. With the aim of describing and discussing the role of ceramides in the stratification of cardiovascular diseases, this narrative review contextualizes the importance of this biomarker in the present cardiology scenario.

## Introduction

Data from the World Health Organization (WHO) show that, out of the 50 million deaths recorded in the last decade, cardiovascular diseases (CVD) were responsible for a significant percentage: around 17 million people.^1^ This mortality is particularly high in the acute phase following an acute myocardial infarction (AMI), with a

10%–15% recurrence of ischemic events within a year and cumulative rates of up to 50% in 10 years.^2^

Approximately 50% of the patients who undergo primary percutaneous coronary intervention have multivessel disease, usually manifested as a progressive chronic condition with high mortality rates. Currently, it is not possible to precisely prevent the recurrence of acute ischemic events, clearly demonstrating the need for biomarkers that may predict coronary atherosclerotic plaque instability.^3^

Recent studies have highlighted the pathophysiological role of classes of lipids other than LDL-cholesterol in atherosclerosis and AMI, including ceramides, sphingomyelin, phosphatidylcholines, and cholesterol esters.^4,5^ Ceramides participate in multiple pathways involved in cellular damage signaling, liberating proinflammatory cytokines that directly modulate apoptosis via the expression of proapoptotic proteins.^6^

Our group has worked, throught mass spectometry, among other molecular techniques, for developing a plasma biomarker capable of diagnosing atherosclerotic plaque instability and predicting reinfartion and progression to Heart Failure (HF) in patients with Acute Coronary Syndrome (ACS).

Ceramides are lipid biomarkers with an emerging role in early diagnosis and risk stratification, acting as marker of primary and secondary cardiovascular (CV) events in patients with clinical and subclinical atherosclerosis who are susceptible to acute ischemic events.^5,7^

### Ceramide: a brief review of the physiology of this new lipid biomarker

Ceramides and LDL-cholesterol are structural lipids that maintain membrane fluidity and integrity through the formation of selective pores, modulating the movement of compounds between intra and extracellular spaces. Ceramides are sphingolipids formed by a sphingosine molecule and a fatty acid, being key components in the formation of cellular membranes^8^ and acting as an

important signaling intermediate in processes that regulate cell homeostasis, such as inflammation, apoptosis, and cellular stress response.^9^

Ceramides accumulate in the coronary atheroma,^10^and their glycosylated forms glucosylceramides and lactosylceramides (Figure 1) are abundant in the developing plaque.^11,12^ Moreover, data from our group showed that the myocardial tissue itself may produce ceramides in a direct response to ischemia and reperfusion.^13^

**Figure 1:** – Sphingolipid metabolic pathways.

Ceramidase is an enzyme that cleaves fatty acids from ceramide for producing sphingosine, which is in turn phosphorylated by sphingosine-1-phosphatase for producing sphingosine-1-phosphate. Ceramide synthesis may occur through the hydrolysis of sphingomyelin (degradation), the salvage (recycling) pathway where sphingosine is phosphorylated, or the de novo pathway where dihydroceramides are desaturated.^9^

### Ceramides: a link between atherosclerosis, diabetes, and dyslipidemia

Ceramides constitute approximately 30% of circulating LDL-cholesterol. An increase in ceramide concentration alters cell membrane permeability, enabling the accumulation of LDL-cholesterol on blood vessel walls. This build-up amplifies the inflammatory process on vessel walls, promoting the apoptosis of vascular smooth muscle cells and endothelial dysfunction, which leads to atherosclerotic plaque instability and rupture^14^ (Figure 2).

**Figure 2:** – Plasma ceramides and plaque rupture. Source: author’s collection.

In addition to the atherosclerotic plaque, this build-up occurs in smooth and skeletal muscles, interfering with the expression of glucose transporter type 4 (GLUT4); this causes a defect in muscle glucose uptake and hinders glycogen synthesis.^15^ Ceramides also stimulate the

apoptosis of pancreatic β cells, directly reducing insulin production.^16^Major et al.^17^ demonstrated that ceramides can mimic the cytotoxic effects of TNF-α, IL-1β, and IFN-γ in pancreatic β-cells, triggering inflammation and apoptosis.^17^ Ceramide accumulation in tissues results in metabolic dysfunction in multiple organs and diabetes complications. Figure 3 shows the main tissues affected by ceramides.^18,19^

**Figure 3:** – Effect of ceramides on different organs.

Therefore, the measurement of these molecules helps the physician determine not only the degree of dyslipidemia and risk of atherosclerosis through LDL-cholesterol levels, but also insulin resistance and β-cell apoptosis through ceramide levels.

Clinical implications led to a patent of this biomarker in the United States and Europe, being currently available for clinical use at referral hospitals such as Mayo Clinic.^20^

### Are screening examinations necessary for the follow-up of subclinical atherosclerosis?

There is a significant gap in the detection of subclinical atherosclerosis and the cost-effective triage and follow-up of this entity, with various noninvasive tests being routinely requested in populations at different levels of CV risk. This is a reality worldwide, and despite the increasing advances in noninvasive tests for detecting atherosclerosis, risk stratification remains imperfect.^21^ Considering the escalating costs of health care, this stereotyped medical practice should thus be reevaluated.

The association of clinical and imaging data for supporting the development of protocols that incorporate classical risk scores such as Framingham and Interheart has been proposed for improving CV risk stratification.

However, these methods, when applied to the general population without a triage step, have limited capacity for assessing CV risk due to aspects related to logistics and costs.^22^

The Multi-Ethnic Study of Atherosclerosis (MESA) study, one of the most relevant in this area, brought important contributions for comprehending the development and progression of CVD from subclinical to clinical stage. In a sub analysis of the MESA study, a large protein biomarker panel was evaluated in the search for predictive markers of CVD progression. When compared to classical CV risk factors, protein biomarkers with various specific panels for inflammation, insulin resistance, lipids, hemostasia, fibrinolysis, oxidative damage, and endothelial stress, among others, had incremental predictive values for long-term adverse CV events that were only borderline (area under the receiver operating characteristic curve [AUC]: 0.768 vs 0.776, p = 0.003) and did not reach an incremental predictive value in the prediction of medium-term adverse CV events (AUC: 0.795 vs 0.796, p = 0.627). Considering these similar ROC curve values, new screening and risk stratification methods are thus required to improve early detection of plaque instability.^23^

Once developed, such a biomarker would enable interventions at the beginning of atherosclerotic plaque progression, being essential for avoiding the large additional costs of atherosclerosis’ symptomatic stage. These considerations are important and should be evaluated when developing platforms for population health screening and seeking adequate cost-effectiveness.

### Should we measure ceramides and LDL-cholesterol?

Subclinical atherosclerosis precedes most CV events, and its detection may improve CV risk stratification.^11,12^ However, an incompatibility has been reported between apparently benign conventional risk factor profiles and subclinical atherosclerosis detected by coronary artery calcification (CAC) or intima thickness measured at the carotid ultrasound.^13,14^

Studies have identified subclinical atherosclerosis in almost 60% of middle-aged individuals at low CV risk according to traditional risk scores, and multiple affected vessel sites were found in 41% of these individuals.^15^ These findings suggest that variables other than conventional CV risk factors may play relevant roles in atherogenesis.

Patients with atherosclerosis consist in a very heterogeneous population, with complex risk stratification, and should not be all considered as being at similar risk for acute events. The use of CV risk scores is currently recommended by guidelines as a stratification tool; however, due to limitations in predictive precision especially in patients at high CV risk, the optimization of risk tools by recalibrating scores or their association with biomarkers is frequently needed for a higher predictive accuracy in different populations.

LDL-cholesterol is a risk factor directly involved in the development of atherosclerotic plaque, therefore being an important therapeutic target in clinical practice. Dieting, lifestyle changes, and use of medications can result in significant and sustained reductions in plasma LDL-cholesterol levels. However, CVD are still one of the main causes of death worldwide,^24^ suggesting that conventional LDL-cholesterol control is not enough; the early detection and prevention of atherosclerotic plaque instability may thus open the way for significantly reducing disease progression.

Current guidelines endorse LDL-cholesterol control and the measurement of nonspecific inflammatory markers such as C-reactive protein (CRP) in CV risk stratification. However, the pathophysiology of atherosclerosis involves the complex intersection of dyslipidemia, inflammation, endothelial dysfunction, platelet activation, and other factors.^25^ Recent data demonstrate possible associations between each of these pathways and plasma ceramide levels, indicating an association and plausible causality of this biomarker in acute CVD and atherosclerotic plaque instability.

Moreover, plasma LDL-cholesterol does not precisely predict major adverse cardiac and cerebrovascular events (MACCE), requiring a thorough medical assessment and a series of noninvasive tests in clinical practice for following up on the progression of atherosclerotic plaques.^26^ On the other hand, plasma ceramide concentrations are increased in various conditions related to cardiac diseases, in addition to their biochemical role in the progression of atherosclerosis, which has also been studied by our group. Preliminary studies have showed an increase in ceramides in the acute phase of AMI and in vulnerable plaques in humans, correlating these findings with pre-clinical data that showed the upregulation of ceramide-producing enzymes in the myocardium, with an increase in plasma ceramide levels within the first 24 hours of an acute ischemic event.^5^

Ceramides are also associated with a higher risk of disease progression in patients with HF. Among 423 patients with acute HF, plasma ceramide concentrations were independently associated with death and worsening of left ventricular function during hospitalization.^27^

### Searching for evidence: ceramides and CV risk prediction

#### Primary prevention:

The FINRISK study was performed in patients with no previous history of CV events and showed that circulating levels of specific ceramides (16:0, 18:0, and 24:1) were significantly associated with subsequent major CV events when compared to individuals who remained asymptomatic. Significant univariate associations between ceramides and fatal events suggest they play a fundamental role in atherosclerotic plaque rupture in this population.^7^

Still considering primary prevention, Petterson et al.^28^demonstrated that the proportion of ceramides C24:0/C16:0 and the plasma concentration of ceramide C24:0 were inversely associated with coronary risk factors such as age and smoking habits, in addition to the development of CAD and HF.^28^

#### CAD:

In a post-hoc analysis of the Systolic Blood Pressure Intervention Trial (SPRINT) study (N = 9631), Nguyen et al. demonstrated that plasma LDL-cholesterol levels were not associated with the primary composite outcome (myocardial infarction, stroke, acute decompensated HF, and CV death).^29^ In addition, when evaluating patients in secondary prevention of CV events (N = 1562), they observed that LDL-cholesterol was only marginally associated with the incidence of CV events (adjusted hazard ratio 1.005 [95% confidence interval, CI = 1.002–1.009], p = 0.005 [1 mg/dl increase], with poor discrimination for MACE [AUC = 0.54, p = 0.087]).^29^

Although studies with PCSK9 inhibitors such as Odyssey Outcomes and Fourier^30^ reinforce the “the lower, the better” principle, meaning there is an association between low LDL-cholesterol levels and a low risk of clinical atherosclerosis, no perfect risk correlation is observed. In order to fill this gap, the measurement of metabolites with metabolomic techniques has been increasingly employed since it presents advantages over classical methods such as a more comprehensive analysis and the acquisition of a metabolic profile of the target tissue considering the disease of interest.

An undirected metabolomic analysis identified 3 plasma ceramides that were significantly associated with CV mortality in a cohort with angiography-confirmed CAD. Ceramides associated with high CV mortality in this study were C16:0, C18:0, and C24:1, and their association was independent from age, body mass index (BMI), smoking habits, use of statins, triglycerides, LDL-cholesterol, and total cholesterol.^14^

Kaasenbrood et al.,31 using the Secondary Manifestations of Arterial Disease (SMART) risk score, attempted to improve the prediction of acute events in this group of patients.^31^ This risk score comprehends clinical and laboratory variables (total cholesterol, HDL-cholesterol, estimated glomerular filtration rate [eGFR], CRP) and was tested in various cohorts; based on the obtained results, the authors suggested new algorithms for estimating CV risk in order to individually and more precisely stratify this population, demonstrating the possibility of improving risk scores by incorporating biomarkers.^31^

In the Prevención con Dieta Mediterránea (Mediterranean Diet Prevention, PREDIMED) study, a prospective cohort study with patients at high CV risk, ceramides C24:0, C22:0, and C16:0 were associated with CVD.4 The odds radios comparing the extreme quartiles of plasma ceramides C16:0, C22:0, C24:0, and C24:1 were 2.39 (1.49–3,83; p < 0.001), 1.91 (1.21–3.01; p = 0.003), 1.97 (1.21–3.01; p = 0.004), and 1.73 (1.09–2.74; p = 0.011), respectively. In another prospective study with approximately 500 patients who underwent elective coronary angiography, Meeusen et al. reported that plasma C16:0, C18:0, and C24:1 levels were independently associated with increased risk of MACCE in a mean follow-up of 4 years.32 The risk associated with ceramides was also independent from traditional risk factors, including age, sex, BMI, smoking habits, and cholesterol. Moreover, the predictive value remained significant after additional adjustments for serum glucose and family history of CAD. These results suggest that, when plasma ceramide levels are high in patients with or without significant stenosis of the coronary artery, the risk of death is high in both groups.^32^

Another risk score involving ceramides is the Coronary Event Risk Test (CERT2), which was developed in the Western Norway Coronary Angiography Cohort (WECAC) study and validated by the Long-Term Intervention with Pravastatin in Ischaemic Disease (LIPID) and Langzeiterfolge der Kardiologischen Anschlussheilbehandlung (KAROLA) studies.^33^ Results showed that the CV risk estimation tool incorporating ceramide measurement could reliably stratify MACE in patients with stable CAD. Additional evidence obtained by the WECAC and LIPID studies demonstrated that these biomarkers alone were able to accurately stratify primary CV risk in patients with and without diabetes; in individuals with diabetes, the only significant predictors in this study were the CERT2 score and high-sensitivity troponin.

#### ACS

In the European Collaborative Project on Inflammation and Vascular Wall Remodeling in Atherosclerosis - Intravascular Ultrasound (favor verificar se está correto.) (ATHEROREMO-IVUS) study (N = 600 patients), Cheng et al. demonstrated that plasma C16:0, C18:0, and C24:1 levels were significantly associated with vulnerable coronary plaque in individuals with ACS. Increased plasma levels of these ceramides were also significantly associated with higher angiographic severity of coronary stenosis,^14,34^ as well as a lower perfusion of the myocardial wall after stress in patients with established or suspected CAD who underwent myocardial perfusion scintigraphy.^35,36^ These findings suggest causality between increased ceramides in the atherosclerotic plaque and its instability or severity.

Using optical coherence tomography (OCT) in patients with ST‐segment–elevation myocardial infarction (STEMI), Bo Yu et al. recently observed increased plasma C16:0, C18:0, and C24:0 levels when compared to individuals with no coronary disease or with stable CAD (p < 0.001, p < 0.001, p < 0.001, p < 0.001, respectively). This was the first study using OCT that proved a positive independent association between plasma ceramide concentrations and plaque rupture, suggesting that plasma ceramide concentrations may act as potential biomarkers of plaque rupture.^37^

Additional evidence of this association was obtained by Laasksonen et al.^5^ in a prospective cohort of patients with stable CAD; increased serum ceramide levels were observed in 81 out of 1580 patients (Table 1), who later presented MACE in 4.6 years of follow-up. This proportion was maintained even after adjustment for treatment with statins. Ceramides were predictive in both cases, with comparable odds ratios (OR) in patients using statins or not: 1.68 (1.31–2.15) vs 1.7 (1.33–2.17). In this study, plasma LDL-cholesterol was not significantly predictive of MACE.^5^

**Table 1:** – Relative risk scores involving ceramides in different cohorts

Score	Category	BECAC (5-year risk)^5^			SPUM-ACS (1-year risk)^5^		

		Deaths (n)	%	Relative Risk	Morte (n)	%	Relative Risk
0-2	Low	15/549	2.7%	1.0	9/575	1.6%	1.0
3-6	Moderate	29/601	4.8%	1.8	16/611	2.6%	1.7
7-9	Increased	20/288	6.9%	2.5	9/270	3.3%	2.1
10-12	Higher	17/149	11.4%	4.2	17/181	9.4%	6.0

*Source: adapted from Mayo Clinic. https://www.mayoclinic.org/*

Laasksonen et al.^5^ analyzed the Special Program University Medicine-Inflammation in Acute Coronary Syndromes (SPUM-ACS) study (N = 1637), performed with patients with ACS, where ceramides were also predictors of MACE regardless of CV risk. In 51 patients who died within a year of a cardiac event, plasma ceramides were found at significantly higher levels when compared to patients who survived during follow-up.^5^

Finally, De Carvalho et al.^13^ assessed patients with AMI in 2 cohorts of patients subjected to invasive stratification, comparing MACCE-free survival rates in high-risk patients as defined by the Global Registry of Acute Coronary Events (GRACE) score adjusted to the local population. In this study, the GRACE score was less capable of predicting event-free survival when compared to an association of 12 plasma ceramides measured in the acute phase of AMI.^13^ This study included Chinese, Malay, and Indian people, ethnicities that represent a considerable proportion of the global population; external revalidation of the predictive value of these biomarkers was performed in a Caucasian population in New Zealand, demonstrating the development of a potentially universal biomarker. These data were also corroborated by the molecular biology analysis of atherosclerotic plaque biopsies obtained from patients subjected to heart surgery who had experienced or not recent infarctions, confirming the increase in ceramide production in patients with vulnerable atherosclerotic plaque.

The main studies evaluating the association between ceramides and risk of acute CV events are demonstrated on Table 2.

**Table 2:** – Main studies evaluating the association between ceramides and risk of acute cardiovascular events (by year of publication)

Author/reference	Study characteristics	Primary outcome	Adjustment	Main result
Laaksonen et al.^5^ European Heart Journal 2016;37, 1967-1976	Prospective cohort study with N = 1580 adults (62 years old; 59% male; BMI 25 kg/m2; LDL-cholesterol 2.8 mmol/l, triglycerides 1.4mmol/l; statin use 62.6%) who underwent elective coronary angiography due to stable CAD and were recruited at the Haukeland University Hospital in Bergen (BECAC study) with 4.6 years of follow-up, in addition to 1637 patients (63 years old; 78% male, BMI 26 kg/m2, LDL-cholesterol 2.6 mmol/l, triglycerides 1 mmol/l, statin use 27.2%) with an ACS diagnosis who underwent invasive treatment in 4 Swiss university hospitals (SPUM-ACS study), with 1-year follow-up	Cardiovascular death	Total cholesterol, triglycerides, HDL-cholesterol, LDL-cholesterol, age, sex, smoking habits, previous acute myocardial infarction, diabetes mellitus, hypertension, previous stroke	Cer (d18:1/16:0) and Cer (d18:1/24:1) were associated with an increased risk of cardiovascular death in all cohorts. OR Cer (d18:1/16:0)/Cer(d18:1/24:0) was 4.49 (95% CI, 2.24–8.98), 1.64 (1.29–2.08), and 1.77 (1.41–2.23) for Corogene, SPUM-ACS, and BECAC studies, respectively
Havulinna et al.^7^ Arteriosclerer Thromb Vasc Biol 2016;36: 2424-2430	Populational cohort study with N = 8101 healthy patients (48 years old; 47% male, BMI 26 kg/m2, LDL-cholesterol 3.3 mmol/l, triglycerides 1.3 mmol/l) from FINRISK 2002	Major cardiac and cerebrovascular adverse events	Total cholesterol, HDL-cholesterol, arterial pressure, diabetes mellitus, and smoking habits	Cer (d18:1/16:0), Cer (d18:1/18:0), and Cer (d18:1/24:1) levels were significantly higher in patients with adverse cardiovascular progression when compared to asymptomatic individuals. Serum concentrations of high-risk ceramides predicting cardiovascular death in patients with CAD were also higher in FINRISK MACE cases when compared to asymptomatic individuals, as follows: Cer (d18:1/16:0), Cer (d18:1/18:0), and Cer (d18:1/24:1) 11.4%, 21.3%, and 17.0%, respectively (p < 0.001 for all)
Wang et al.^4^ Circulation 2017; 135: 2028-2040	Cohort study nested in the PREDIMED randomized study with N = 980 participants (68 years old; 45% male, BMI 30 kg/m2, LDL-cholesterol 3.4 mmol/l, triglycerides 1.6 mmol/l), including 230 cases of CVD and 767 randomly selected participants. The sub cohort included 37 overlapping CVD cases. Two participants with undetectable plasma ceramide concentrations were excluded. Follow-up: 4.5 years	MACE	IAge, sex, BMI, family history of premature CAD, smoking habits, history of hypertension, dyslipidemia, and type 2 diabetes.	Among the high-risk ceramides identified, the upper quartiles of plasma Cer (d18:1/16:0), Cer (d18:1/22:0), Cer (d18:1/24:0), and Cer (d18:1/24:1) levels were associated with an adverse cardiovascular outcome. The multivariable hazard ratios comparing the extreme quartiles of plasma C:16, C22:0, C24:0, and C24:1 concentrations were 2.39 (1.49–3.83, p < 0.001), 1.91 (1.21–3.01, p = 0.003), 1.97 (1.21–3.01, p = 0.004), and 1.73 (1.09–2.74, p = 0.011), respectively.
De Carvalho et al.^13^ JACC Basic Transl Sci 2018;3:163-175	Prospective longitudinal study with N = 327 patients from a primary cohort (57 years old; 90% male, BMI 26 kg/m2, LDL-cholesterol 3.1 mmol/l, triglycerides 1.2 mmol/l) and 119 patients in the validation cohort (66 years old; 72% male, BMI 29 kg/m2, LDL-cholesterol 3.2 mmol/l) with ACS who underwent invasive stratification with plasma measurements performed before and after stratification; 1-year follow-up.	Major cardiac and cerebrovascular adverse events	GRACE	Among the high-risk ceramides previously identified, the plasma Cer (d18:1/16:0), Cer (d18:1/18:0), and Cer (d18:1/24:1) levels were associated with adverse cardiovascular events
Meeusen et al.^32^ Arterioscler Thromb Vasc Biol. 2018; 38: 1933-1939	Cross-sectional study: 495 participants (60 years old; 62% male, BMI 28kg/m2, LDL-cholesterol 3.1 mmol/l, triglycerides 1.7 mmol/l, statin use 28.5%) before nonurgent coronary angiography. Follow-up: 4 years	MACE (myocardial infarction, percutaneous intervention, myocardial revascularization surgery, stroke, or death).	Age, sex, BMI, hypertension, smoking habits, LDL-cholesterol, HDL-cholesterol, triglycerides, glycemia, family history of CAD	Among the high-risk ceramides previously identified, plasma Cer (d18:1/16:0), Cer (d18:1/18:0), and Cer (d18:1/24:1) levels were associated with adverse cardiovascular events. Adjusted hazard ratios per standard deviation (95% CI) were 1.50 (1.16–1.93) for Cer (16:0), 1.42 (1.11–1.83) for Cer (18:0), and 1.43 (1.08–1.89) for Cer (24:1)
Peterson et al.^28^ J Am Heart Assoc. 2018;7: e007931	Community-based study: 2642 participants from the Framingham Heart Study (FHS; 66 years old; 46% male, BMI 28 kg/m2, LDL-cholesterol 2.7 mmol/l, triglycerides 1.3 mmol/l, statin use 42.7%) and 3134 participants from the Study of Health in Pomerania (SHIP; 54 years old, 48% male, BMI 28 kg/m2, LDL-cholesterol 5.5 mmol/l, triglycerides 1.8 mmol/l, statin use 14.5%) were followed up for 6 and 8 years, respectively	MACE (fatal and non-fatal cardiovascular events)	Age, sex, BMI, hypertension, diabetes mellitus, smoking habits, anti-hypertensives, total cholesterol/HDL-cholesterol ratio, triglycerides, and lipid-lowering drugs	Among the high-risk ceramides previously identified, only Cer (d18:1/24:0) were associated with adverse cardiovascular outcomes. In the meta-analysis of both cohorts and after adjusting risk factors for CAD, C24:0/C16:0 ratios were inversely associated with CAD (hazard ratio per mean standard deviation increase, 0.79; 95% CI, 0.71–0.89; p < 0.0001) and inversely associated with HF (hazard ratio, 0.78; 95% CI, 0.61–1,00; p = 0.046).
Hilvo et al.^33^ European Heart Journal 2019, in press	Longitudinal study; 3 large cohort studies: 3789 patients (62 years old; 72% male, LDL-cholesterol 2.9 mmol/l, triglycerides 1.5 mmol/l, statin use 72.6%) from the Western Norway Coronary Angiography Cohort (WECAC); 5991 patients (65 years old; 83% male, LDL-cholesterol 3.9 mmol/l, triglycerides 1.6 mmol/l, statin use 49.9%) from the Long-Term Intervention with Pravastatin in Ischaemic Disease (LIPID) study; and 1023 patients (62 years old; 84% male, LDL-cholesterol 3 mmol/l, triglycerides 1.6 mmol/l, statin use 75.6%) from the Langzeiterfolge der Kardiologischen Anschlussheilbehandlung (KAROLA) study. Follow-up: 6 years	MACE (composite endpoint including death due to CV events, MI, and stroke )	IAge, sex, treatment with statins (WECAC, KAROLA), diabetes mellitus, hypertension, current smoking habit, previous MI, previous stroke, stratified according to vitamin B intervention (WECAC) and treatment group (LIPID).	A simple risk score, based on ceramides and phosphatidylcholines with the best prognostic characteristics, was developed by the WECAC study and validated in the other 2 cohorts. This score was highly significant for predicting mortality due to CVD (multi-adjusted hazard ratios [95% CI] per standard deviation were 1.44 [1.28–1.63] at the WECAC, 1.47 [1.34–1.61] at the LIPID study, and 1.69 [1.31– 2.17] at the KAROLA study). Moreover, a combination of the risk score with high-sensitivity troponin T increased hazard ratios to 1.63 (1.44–1.85) and 2.04 (1.57–2.64) in the WECAC and KAROLA cohorts, respectively.

*BECAC: Bergen Coronary Angiography Cohort; DAC: doença arterial coronariana; FINRISK: population-based risk factor survey ; IC95%: intervalo de confiança de 95%; KAROLA: Langzeiterfolge der KARdiOLogischen Anschlussheilbehandlung; LIPID: Intervenção a Longo Prazo com Pravastatina em Doença Isquêmica; MACCE: eventos adversos cardíacos e cerebrovasculares maiores; MACE: eventos adversos cardíacos maiores; RR: razão de risco; SPUM-ACS: Special Program University Medicine-Inflammation in Acute Coronary Syndromes ; WECAC: The Western Norway Coronary Angiography Cohort.*

## Conclusion

Plasma ceramides are elevated in patients with MACCE, and pre-clinical and clinical studies demonstrate an association between these lipids and atherosclerotic plaque instability.

Their measurement has incremental value for risk stratification, in addition to the classic risk factors both in primary and secondary CV prevention; consecutive measurements may have higher incremental predictive value than other biomarkers considering future adverse events. However, we still need further evidence from randomized studies to assess the impact of this marker on prognosis and of treatment escalation guided by plasma ceramide levels.
